# Modulating the Electron Transporting Properties of Subphthalocyanines for Inverted Perovskite Solar Cells

**DOI:** 10.3389/fchem.2022.886522

**Published:** 2022-06-14

**Authors:** Jorge Labella, Cristina Momblona, Pavel Čulík, Elisa López-Serrano, Hiroyuki Kanda, Mohammad Khaja Nazeeruddin, Tomás Torres

**Affiliations:** ^1^ Departamento de Química Orgánica, Universidad Autónoma de Madrid, Madrid, Spain; ^2^ Group for Molecular Engineering of Functional Materials, Institute of Chemical Sciences and Engineering, École Polytechnique Fédérale de Lausanne (EPFL Valais Wallis), Sion, Switzerland; ^3^ Institute for Advanced Research in Chemical Sciences (IAdChem), Universidad Autónoma de Madrid, Madrid, Spain; ^4^ IMDEA-Nanociencia, Campus de Cantoblanco, Madrid, Spain

**Keywords:** subphthalocyanines, non-fullerene acceptor, photovoltaics, perovskite solar cells, electron-transport materials

## Abstract

The lack of organic non-fullerene ETMs with good electron transport and device stability is an important problem for the further development and commercialization of perovskite solar cells. Herein, the use of SubPcs as ETMs in PSCs is explored. To this end, we analyze the influence of SubPc peripheral functionalization on the efficiency and stability of *p-i-n* PSCs. Specifically, ETMs based on three SubPcs (with either six or twelve peripheral fluorine and chlorine atoms) have been incorporated into PSCs with the perovskite layer deposited by solution processing (CsFAMAPbIBr). The device performance and morphology of these devices are deeply analyzed using several techniques, and the interfacial effects induced by the SubPcs are studied using photoluminescence and TR-PL. It is observed that the device stability is significantly improved upon insertion the SubPc layer. Moreover, the impact of the SubPc layer-thickness is assessed. Thus, a maximum power conversion efficiency of 13.6% was achieved with the champion device.

## Introduction

Due to their excellent properties, low cost, and facile preparation (Heo et al., 2013; Hodes, 2013), organic-inorganic metal halide perovskite solar cells (PSCs) have witnessed an unprecedentedly extensive research activity over the past decade (Snaith, 2018; Lu et al., 2020; Vasilopoulou et al., 2020; Li et al., 2021). Thus, within only a few years, PSCs have reached impressive power conversion efficiency (PCE) values of up to 25.5% by employing the conventional *n-i-p* configuration (Best Research-Cell Efficiency Chart, 2021). However, the cost-effective mass production and flexible applications of PSCs still remain problematic since, in such *n-i-p* devices, the metal oxide layer (typically TiO_2_) suffers from low stability under UV irradiation and requires high-temperature treatments (Leijtens et al., 2013; Di Giacomo et al., 2016). Consequently, inverted PSCs, also known as *p-i-n* devices, have recently piqued special attention within the solar cell community since they present more advantageous properties, such as low-temperature processing, the potential to construct tandem solar cells, negligible hysteresis, and compatibility with printing technology (Lin et al., 2020). Nonetheless, despite showing these features, the PCEs of *p-i-n* PSCs still lag behind in comparison with those of the *n-i-p* configuration. Such a gap in efficiency mainly stems from the recombination losses ascribed to defects on the perovskite (PVK) surface and at the grain boundaries, and the mismatched energy-level between the PVK and the fullerene-based electron transport material (ETM) (Xu et al., 2015; Luo et al., 2020; Wu et al., 2020), which, in addition, exhibit additional shortcomings such as intrinsic degradation by light-induced dimerization, extrinsic degradation by oxygen and water, and high-energy inputs in their synthesis, purification, and functionalization (Gil-Escrig et al., 2016; Zhao et al., 2016; Said et al., 2019; Zhang et al., 2019). As a result, the search for non-fullerene acceptors (NFA) that simultaneously improve the PVK stability/morphology, exhibit good stability against light and moisture, and display tunable energy levels has become a hot topic in the field of PSCs.

**FIGURE 1:**
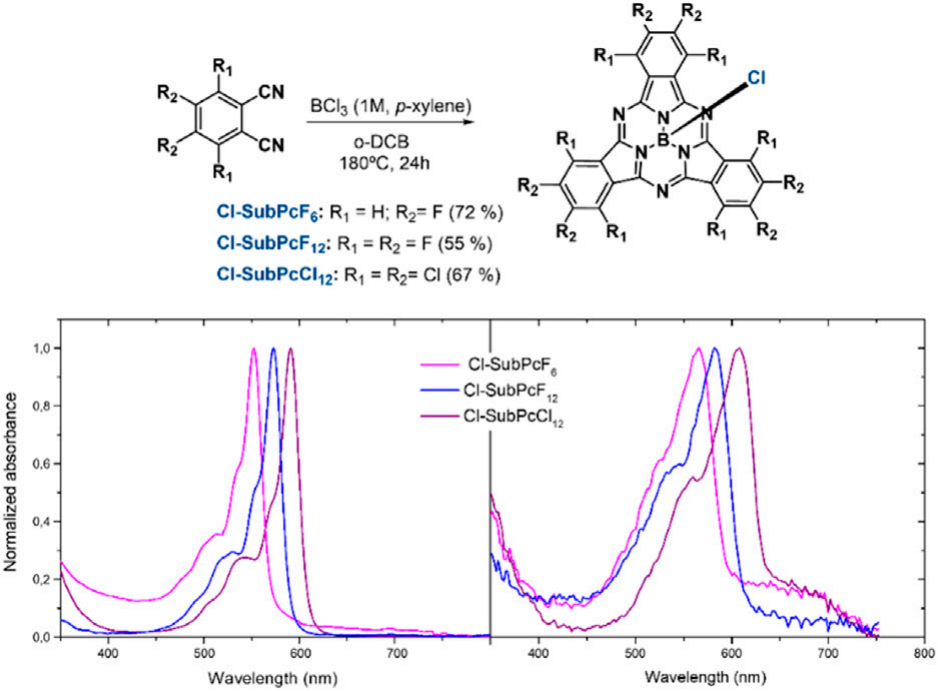
Synthetic route and UV/Vis absorbance spectra in solution (THF) (right) and in a thin film (left) of **Cl-SubPcF**_**6**_, **Cl-SubPcF**_**12,**_ and **Cl-SubPcCl**_**12**_.

Porphyrinic macrocycles, such as phthalocyanines (Pcs) or porphyrins (Ps), currently represent a fundamental family of compounds for the construction of organic solar cells (OSCs) (Imahori and Fukuzumi, 2004; Walter et al., 2010; Li and Diau, 2013). In this sense, given the donor properties of these materials, Pcs and Ps are typically employed as *p*-type semiconductors (Urbani et al., 2019). However, when changing to their contracted homologues, such as Subphthalocyanines (SubPcs; Figure 1), porphyrinic materials can become excellent acceptors if appropriately functionalized (Claessens et al., 2014). Indeed, SubPcs peripherally decorated with electron-withdrawing groups (*i.e.,* halogen atoms or diimides) actually hold a privileged position among the most versatile and promising *n*-type semiconductors by producing excellent PCEs in a variety of fullerene-free solar cells, ranging from planar- and bulk-heterojunction to tandem solar cells (Cnops et al., 2014; Cnops et al., 2015; Duan et al., 2017; Huang T. et al., 2019; Huang X. et al., 2019; Cai et al., 2020).

Despite having these precedents and observing that SubPcs are known to actively interact with the perovskite layer, forming strong Pb-halide bonds, the use of SubPcs as ETMs is still an unexplored territory that, just very recently, has started to be investigated (Chen et al., 2021; Labella et al., 2021). Indeed, our groups have reported that a double-layer ETM consisting of a hexachlorinated SubPc and C_60_ leads to PCEs of up to 10.8%. In this work, the influence of the substituent at the boron atom (*i.e.,* chlorine or fluorine) and the processing method of the PVK layer on the device performance and stability was analyzed. It was observed that the axial chlorine atom and solution-processed triple cation PVK (CsFAMAPbIBr) are the best options as they furnish higher efficiencies and stabilities. However, we unfortunately observed that the SubPc did not efficiently extract the charges from the PVK layer, possibly due to an insufficiently low lowest unoccupied molecular orbital (LUMO) level or to a non-optimal supramolecular organization of SubPc molecules.

With the aim of improving the device performance and further understanding the structure-efficiency relationship of SubPc-based ETMs in PSCs, herein we analyze the influence of SubPc peripheral functionalization on the efficiency and stability of *p-i-n* PSCs. For this purpose, we prepared and evaluated three different SubPcs (Figure 1) with varying electronic and supramolecular properties. In particular, we synthetize SubPcs functionalized with either six or twelve peripheral fluorine or chlorine atoms (**Cl-SubPcF**_**6**_, **Cl-SubPcF**_**12,**_ and **Cl-SubPcCl**_**12**_), since it has been previously demonstrated that they present good supramolecular organization and low-lying LUMO levels (Bukuroshi et al., 2021). In combination with C_60_, these SubPc materials are then implemented as ETM in PSC devices based on a solution processed PVK (CsFAMAPbIBr) active layer. The PV performance of the studied PSCs is deeply characterized by current density–voltage (*J–V*) curves, external quantum efficiency (EQE), and maximum power point tracking (MPPT). In order to understand the SubPc structure’s influence on the device stability, water contact angle measurements in PVK/SubPc layers are also performed. Moreover, the morphology is observed by scanning-electron-microscopy (SEM) and the electron extraction properties of these novel ETMs are analyzed by steady-state photoluminescence (PL) and time-resolved photoluminescence (TRPL). Finally, the impact of SubPc layer-thickness is also assessed for the SubPc leading to the best efficiency. Thus, we achieved a maximum PCE of 13.6% with the best performing device, which, in addition, showed better stability than devices containing only C_60_ as ETM.

## Results and Discussion

### Synthesis and Characterization of SubPc Materials

As shown in Figure 1, **Cl-SubPcF**_**6**_, **Cl-SubPcF**_**12,**_ and **Cl-SubPcCl**_**12**_ were synthesized from commercially available phthalonitrile precursors by cyclotrimerization. Such a reaction was accomplished following a previously reported method developed in our labs for the preparation of halogenated SubPcs (Labella et al., 2021). Thus, **Cl-SubPcF**_**6**_, **Cl-SubPcF**_**12,**_ and **Cl-SubPcCl**_**12**_ were obtained in a 72, 55, and 67% yield, respectively, after simple column chromatography purification. These derivatives exhibited good solubility in organic solvents as well as excellent stability against light, ambient moisture, and oxygen.

**FIGURE 2:**
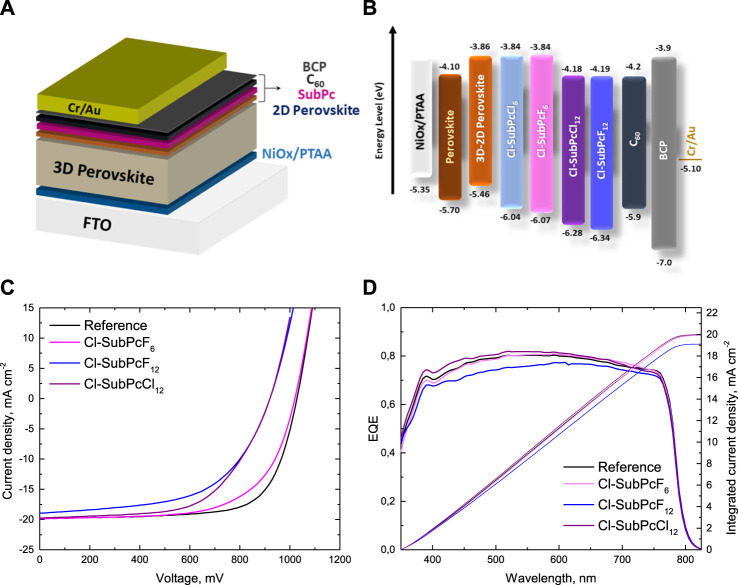
*p-i-n* solar cells containing **SubPc/C**_**60**_ as the ETL. **(A)** Scheme of the perovskite solar cell structure. **(B)** Energy level diagram of the device. **(C)** *J*–*V* curves and **(D)** EQE spectra and the corresponding integrated short circuit current of the most efficient devices containing 2 nm **Cl-SubPcF**_**6**_, **Cl-SubPcF**_**12**_ and **Cl-SubPcCl**_**12**_. The *J–V* curve and the EQE spectrum of the control device (black line) were added for comparison.

The aggregation properties of **Cl-SubPcF**_**6**_, **Cl-SubPcF**_**12,**_ and **Cl-SubPcCl**_**12**_ at the solid state were preliminary analyzed by comparing their absorbance spectra both in solution and in thin film configuration. As shown in Figure 1, in the solid state, all SubPcs display the expected Q band which, although experiencing a slight broadening, remains similar in shape in comparison with those of in-solution spectra. Nevertheless, a ∼15–20 nm red-shift in the absorption maxima is observed, which points out to the formation of columnar H-type-like aggregates in the solid state as a result of the SubPc bowl-shape and dipole moment (Guilleme et al., 2015; Mayoral et al., 2020; Zhang et al., 2020). Then, in order to investigate the electronic properties of SubPcs, their oxidation and reduction potentials were determined in THF by cyclic voltammetry (CV) using 0.1 M tetrabutylammonium hexafluorophosphate (TBAPF_6_) as an electrolyte and ferrocene (Fc/Fc^+^) as the internal reference (See SI). Employing the equation described by Bazan *et al.* (Cardona et al., 2011), the LUMO energies (Figure 2B) were calculated for **Cl-SubPcF**_**6**_, **Cl-SubPcF**_**12,**_ and **Cl-SubPcCl**_**12.**_ As expected, all SubPcs provided a good energy-alignment with the PVK layer. As a result of the higher number of peripheral halogen atoms, both **Cl-SubPcF**_**12**_ and **Cl-SubPcCl**_**12**_ exhibit the lowest LUMO energies (-4.19 eV and -4.18 eV, respectively), which is similar to that of C_60_ (-4.20 eV). In contrast, **Cl-SubPcF**_**6**_ exhibits a LUMO energy value of -3.89 eV, which is similar to that of the hexachlorinated SubPc (hereafter referred to as **Cl-SubPcCl**_**6**_) employed in our previous work (LUMO energy of -3.84 eV). (Labella et al., 2021).

### Device Performance and Characterization

In order to evaluate the function of **Cl-SubPcF**_**6**_, **Cl-SubPcF**_**12,**_ and **Cl-SubPcCl**_**12**_ as ETMs, inverted PSCs were fabricated with the device structure of FTO/NiOx/PTAA/CsFAMAPbIBr/PEAI/SubPc/C_60_/BCP/Cr/Au (Figure 2A), where NiOx and poly (triarylamine) (PTAA) were employed as hole transporting materials (HTMs) (Lian et al., 2020) and bathocuproine (BCP) was used to ensure the ohmic contact between C_60_ and the top electrode (Chen et al., 2017). In addition, a 1 nm-thin layer of Cr was deposited to improve the device stability in air (Kaltenbrunner et al., 2015). In such devices, the triple cation PVK layer was deposited following an experimental procedure previously reported by some of us, and phenethylammonium iodide (PEAI) was further added to form a low-dimensional 2D PVK to improve the PVK surface and stability (Daskeviciute et al., 2021). According to our previous work, the ETM is based on a SubPc/C_60_ double-layer deposited by thermal evaporation, with 2 nm being the optimal thickness for the SubPc material. The solar cells using only C_60_ as a single ETL (*i.e.*, 0 nm of SubPc material) were taken as a reference device.

Figure 2 illustrates the current density–voltage (*J–V*) characteristics (under AM 1.5G irradiation at 100 mWcm^−2^) of the champion devices using **Cl-SubPcF**_**6**_, **Cl-SubPcF**_**12,**_ and **Cl-SubPcCl**_**12**_ as ETMs. All relevant photovoltaic parameters including open-circuit voltage (*V*_**OC**_), short-circuit current (*J*_SC_), and fill factor (*FF*) of the devices are collected in Table 1. Moreover, the PCE distribution based on eight devices per device condition is shown in Supplementary Figure S1. As observed in Figure 1C, the photovoltaic performance of PSCs is strongly affected by the peripheral substituent, being **Cl-SubPcF**_**6**_ the best-performing SubPc material by showing a PCE of 13.0%, with a remarkable *V*_OC_ of 1020 mV, a *J*_SC_ of 19.89 mA cm^−2^, and a *FF* of 0.64. By contrast, **Cl-SubPcF**_**12**_ and **Cl-SubPcCl**_**12**_ showed lower PCEs values of 9.9% and 10.9%, respectively, mainly caused by the drop of *V*_OC_ values (920 mV), which might be attributed to a higher recombination at the perovskite/SubPc interface. Importantly, **Cl-SubPcF**_**6**_ exhibits similar *V*_OC_ and *J*_SC_ but lower FF values than the reference, which furnished PCEs of about 14.5%. Importantly, it should be noted that **Cl-SubPcF**_**6**_, **Cl-SubPcF**_**12,**_ and **Cl-SubPcCl**_**12**_ exhibited higher *J*_SC_ values compared to that of previously tested **Cl-SubPcCl**_**6**_ (Table 1), suggesting that these novel SubPc materials extract the charges more efficiently from the PVK. Further insights into *J*_SC_ were provided by recording the external quantum efficiency (EQE) curves (Figure 2D), whose integrated *J*_SC_ values match well to the *J*_SC_ values obtained from the *J–V* curve under AM 1.5G. In line with *J–V* curves, the EQE spectra show that the photoresponse from 350 to 800 nm is higher for **Cl-SubPcCl**_**12**_ than for **Cl-SubPcF**_**12**_ and **Cl-SubPcF**_**6**_, indicating the improved electron collection of **Cl-SubPcCl**_**12**_ in comparison with their F-based SubPc analogues.

**TABLE 1:** PV parameters of the most efficient devices extracted from the corresponding *J*–*V* curves. *FWD*: forward scan (from *J*_**SC**_ to *V*_**OC**_), *REV*: reverse scan (from *V*_**OC**_ to *J*_sc_).

ETM	Scan Direction	*V*_OC_ (mV)	*J*_SC_ (mA Cm-2)	FF	PCE (%)
Reference (C_60_)	*REV*	1037	19.90	0.69	14.2
	*FWD*	1028	19.88	0.71	14.5
**Cl-SubPcF**_**6**_	*REV*	1024	19.89	0.62	12.6
	*FWD*	1020	19.89	0.64	13.0
**Cl-SubPcF**_**12**_	*REV*	933	18.98	0.54	9.6
	*FWD*	920	18.94	0.57	9.9
**Cl-SubPcCl**_**12**_	*REV*	920	19.73	0.60	10.9
	*FWD*	920	19.75	0.60	10.9
**Cl-SubPcCl**_**6**_ a	*REV*	997	17.39	0.57	9.9
	*FWD*	987	17.38	0.63	10.8

a PV, parameters previously reported in reference 13b.

**FIGURE 3:**
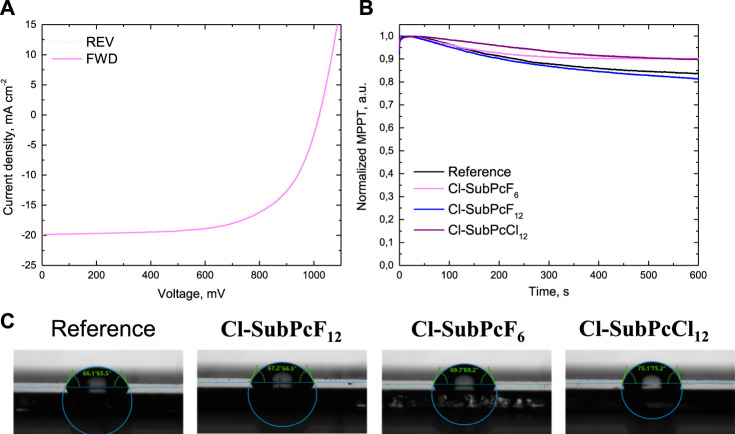
**(A)** *J–V* curve hysteresis of **Cl-SubPcF**_**6**_. **(B)** stability of the PSCs containing 2 nm-vacuum deposited **Cl-SubPcF**_**6**_, **Cl-SubPcF**_**12**_ and **Cl-SubPcCl**_**12**_ layers, measured under one sun illumination in power point tracking conditions in air. **(C)** Images of the water droplet contact angles on the surfaces of the perovskite (reference) and perovskite/SubPc, Cl-SubPcF_12_, Cl-SubPcF_6_, and Cl-SubPcCl_12_ thin films.

To investigate the hysteresis behavior of PSCs, their PCE were examined in both the forward and the reverse scan directions and the corresponding hysteresis index (HI) values were calculated with the reported formulae (Habisreutinger et al., 2018). As demonstrated in Figure 3A, Supplementary Figure S3 and Table 1, all devices exhibited similar *J–V* regardless of the scan direction, indicating almost negligible hysteresis effect in our devices with HI values of 0.02 (reference), 0.03 (**Cl-SubPcF**_**6**_ and **Cl-SubPcF**_**12**_) and none for **Cl-SubPcCl**_**12.**_ Subsequently, the stability of our devices was evaluated by monitoring the PCE at the maximum power output point as a function of time (Figure 3B). Remarkably, it was observed that the PCE of PSCs containing **Cl-SubPcF**_**6**_ and **Cl-SubPcCl**_**12**_ decreased over time much more slowly than the reference. Thus, it can be concluded that the integration of these SubPcs in the ETM significantly improves the device stability. By contrast, **Cl-SubPcF**_**12**_ showed the lowest device stability, which could be the reason for the low efficiency observed with this material. With the aim of understanding such a stabilization upon the addition of SubPcs, the water contact angles of the PVK/SubPc and PVK reference samples were measured (Figure 3C). Compared with PVK (65.8°) and PVK/**Cl-SubPcF**_**12**_ (66.9°), larger average contact angles of about 69.5° and 75.2° were obtained for the PVK/**Cl-SubPcF**_**6**_ and PVK/**Cl-SubPcCl**_**12**_, respectively. Thus, the more hydrophobic properties of **Cl-SubPcF**_**6**_ and **Cl-SubPcCl**_**12**_ could provide a better physical barrier to block H_2_O and O_2_ from penetrating the underlying PVK layer and contribute to the higher ambient stability of the corresponding PSCs.

**FIGURE 4:**
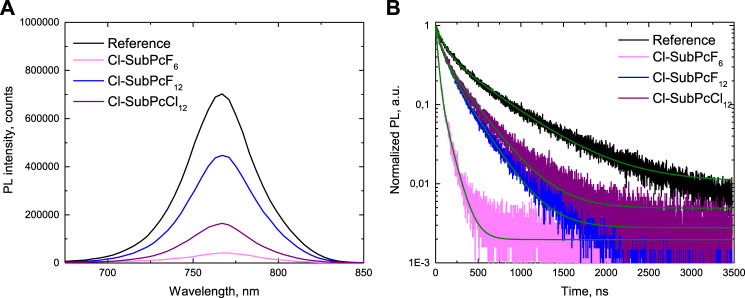
Photoluminescence properties of PVK and PVK/SubPc samples deposited on glass: **(A)** PL spectra (λ_**exc**_ = 450 nm) and **(B)** time-resolved PL spectra (λ_**exc**_ = 640 nm, λ_**em**_ = 780 nm).

Further insights into the electron extraction properties of the developed ETMs were provided by steady-state photoluminescence (PL) quenching measurements of the pristine PVK film and the PVK films covered with **Cl-SubPcF**_**12**_, **Cl-SubPcF**_**6**_, and **Cl-SubPcCl**_**12**_. As shown in Figure 4A, significant quenching of PL emission by SubPc ETMs is observed. In order to evaluate the effect of the evaporated SubPc on the charge transfer, the PL spectra and TRPL decays were measured before (bare perovskite) and after the SubPc deposition (perovskite/SubPc). From this data, the % quenching of each sample was calculated with respect to the corresponding perovskite layer. In particular, **Cl-SubPcF**_**6**_ exhibits the best quenching effect (**see % quenching in** Supplementary Table S1 **),** indicating that **Cl-SubPcF**_**6**_ possesses the most appealing merits for extracting electrons from the PVK. In addition, time-resolved PL (TRPL) decay measurements were conducted to further analyze the carrier dynamics behaviors. As shown in Figure 4B, the TRPL curves exhibited biexponential decays with fast and slow components (Bi et al., 2016). The pristine PVK film has an average lifetime of 99.0 ns. The conformal coating of ETMs on the PVK film promotes the PL decay to 25.8 ns for PVK/**Cl-SubPcF**_**6**_, 70.0 ns for PVK/**Cl-SubPcF**_**12**_ and 55.7 ns for PVK/**Cl-SubPcCl**_**12**_, respectively. Thus, it can be concluded that the carriers generated from the PVK absorber are extracted more efficiently by the **Cl-SubPcF**_**6**_, confirming that the **Cl-SubPcF**_**6**_ ETM has a superior electron transfer property compared to the **Cl-SubPcF**_**12**_ and **Cl-SubPcCl**_**12**_.

**FIGURE 5:**
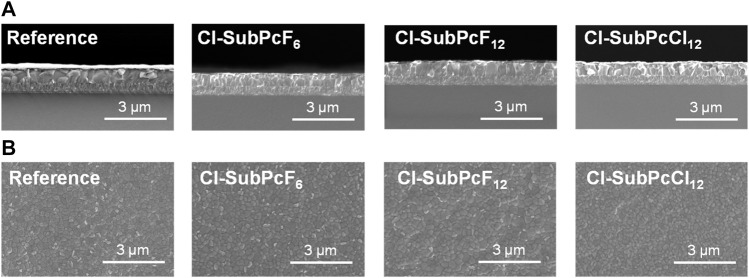
**(A)** Cross-sectional and **(B)** top-view SEM images of the reference and SubPc-containing devices.

The thin film morphology of our devices was studied by scanning electron microscopy (SEM; Figure 5). A cross-sectional SEM image of the completed device (FTO/NiOx/PTAA/PVK/PEAI/SubPc/C_60_/BCP/Cr/Au) is depicted in Figure 5A. For all ETMs, the images show a well-ordered structure where all layers, except the thin transport layers, are visible with a uniform and homogeneous morphology. Similar characteristics are observed in the reference device. On the other hand, top-view scanning electron microscope images were obtained from SubPc films deposited on top of PVK layers (Figure 5B). As shown in the images, the SubPc surface does not show any aggregation of the material, suggesting a conformal coating of the perovskite layer. Therefore, we can assume that the different performances observed with SubPcs **Cl-SubPcF**_**12**_, **Cl-SubPcF**_**6**_, and **Cl-SubPcCl**_**12**_ mainly stem from the electronic features of the SubPc derivatives and not from differences in the morphology.

### Influence of the Cl-SubPcF_6_ SubPc Layer Thickness

**FIGURE 6:**
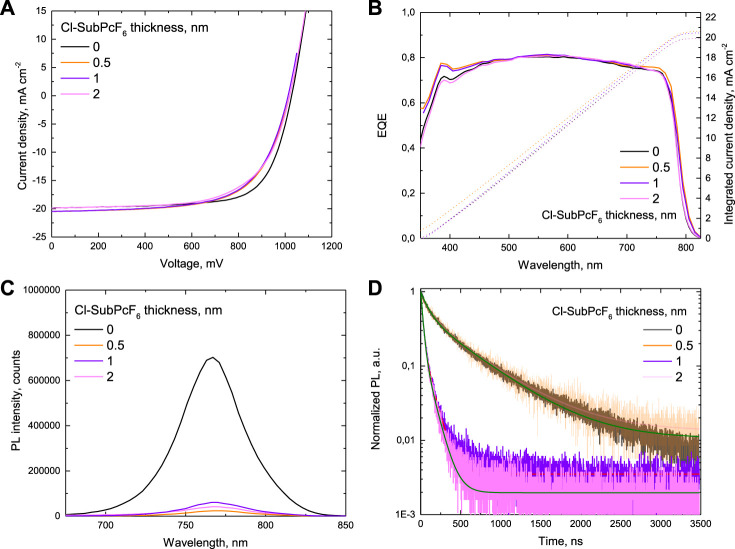
*p-i-n* solar cells containing **Cl-SubPcF**_**6**_ **/C**_**60**_ as the ETM with SubPc layer-thicknesses of 2 nm, 1 nm, and 0.5 nm. **(A)** *J*–*V* curves and **(B)** EQE spectra of the most efficient devices. The *J–V* curve and the EQE spectra of the control device (black line) were added for comparison. **(C)** PL spectra (**λ**_exc_ = 475 nm) and **(D)** time-resolved PL spectra (**λ**_exc_ = 640 nm, **λ**_em_ = 780 nm) of PVK and PVK/**Cl-SubPcF**_**6**_ **(**2 nm, 1 and 0.5 nm) samples.

Once it was demonstrated that **Cl-SubPcF**_**6**_ is the most efficient ETM, we explored whether lowering the SubPc layer thickness could result in an enhancement of the device performance. To this end, we prepared devices similar to those described earlier, but inserting 0.5 nm or 1 nm of SubPc between the PVK layer and C_60_ ETM, instead of 2 nm. The *J*–*V* characteristics and the detailed photovoltaic parameters of these devices are shown in Figure 6 and summarized in Table 2, respectively. Interestingly, the PCE raised up to 13.6% upon decreasing the SubPc layer thickness from 2 to 1 nm and 0.5 nm. These higher efficiencies mainly arise from gains in the *J*_SC_ values, which, surprisingly, exceeded those of the reference device. However, the SubPc-containing devices still suffer from lower *FF* values than the reference. Similar to previous devices, the *J–V* curves recorded in the reverse scan direction reveal that these devices also exhibit negligible hysteresis, being almost 0 in the case of 0.5 nm layer thickness. On the other hand, PL (Figure 6C) and TRPL (Figure 6D) measurements of the PVK film covered 1 and 0.5 nm of Cl-SubPcF_6_ confirmed the excellent ability of SubPc to extract charges by showing a strong quenching effect in the emission of the PVK as well as a shorter PL decay time.

**TABLE 2:** PV parameters of the most efficient devices extracted from the corresponding *J*–*V* curves. *FWD*: forward scan (from *J*_**SC**_ to *V*_**OC**_), *REV*: reverse scan (from *V*_**OC**_ to *J*_**SC**_).

Cl-SubPcF_6_ thickness (nm)	Scan direction	*V*_OC_ (mV)	*J*_SC_ (mA cm^−2^)	FF	PCE (%)
Reference (C_60_)	*REV*	1037	19.90	0.69	14.2
**0**	*FWD*	1028	19.88	0.71	14.5
**0.5**	*REV*	1019	20.47	0.65	13.5
	*FWD*	1006	20.47	0.66	13.6
**1**	*REV*	1013	20.48	0.64	13.3
	*FWD*	1006	20.48	0.66	13.6
**2**	*REV*	1024	19.89	0.62	12.6
	*FWD*	1020	19.89	0.64	13.0

## Conclusion

In summary, three SubPc derivatives, namely **Cl-SubPcF**_**6**_, **Cl-SubPcF**_**12,**_ and **Cl-SubPcCl**_**12**_, have been developed as ETMs in PSCs. For this study, the SubPcs have been incorporated into double-layers with C_60_ in inverted PSCs based on a triple cation solution-processed PVK (CsFAMAPbIBr). **Cl-SubPcF**_**6**_ with the highest LUMO level provided better results than **Cl-SubPcF**_**12**_ and **Cl-SubPcCl**_**12**_. Thus, inverted PSCs using the **Cl-SubPcF**_**6**_ ETM showed a leading efficiency of 13.0%, significantly higher than 10.9% for **Cl-SubPcCl**_**12**_ and 9.9% for **Cl-SubPcF**_**12**_. As deduced from the *J–V* curves and EQE spectra, as well as from the photoluminescence study, the higher performance of **Cl-SubPcF**_**6**_ mainly arises from the excellent charge extraction capability of this material in comparison with that of **Cl-SubPcF**_**12**_ and **Cl-SubPcCl**_**12**_. Importantly, MPPT measurements revealed that the insertion of the **Cl-SubPcF**_**6**_ layer between the PVK and C_60_ significantly improves the device stability. Moreover, it was found that the reduction of SubPc layer thickness from 2 to 1 nm or 0.5 nm further increases the device efficiency, which reaches values of up to 13.6%. The SEM images of our devices also revealed that our fabrication method is very efficient for incorporating a SubPc as the electron transport layer of PSCs.

In light of these results, we strongly believe that SubPc has great potential as non-fullerene ETMs in PSCs and this work establishes fundamental guidelines in this sense. Further research employing novel SubPcs as ETM is ongoing in our laboratories.

## Data Availability

The original contributions presented in the study are included in the article/Supplementary Material; further inquiries can be directed to the corresponding authors.
