# Supplementary material for: Modulating the Electron Transporting Properties of Subphthalocyanines for Inverted Perovskite Solar Cells
Source: Front Chem. 2022 Jun 14;10:886522. doi: 10.3389/fchem.2022.886522 (PMC9329656; doi:10.3389/fchem.2022.886522)
Supplement: Supplementary file 1 [file DataSheet1.docx]

*Supporting Information for*

**Modulating the electron transporting properties of Subphthalocyanines for inverted perovskite solar cells.**

Jorge Labella^1,†^, Cristina Momblona ^2,†^, Pavel Čulík^2^, Elisa López-Serrano^1^, Hiroyuki Kanda^2^, Mohammad Khaja Nazeeruddin^2*^, Tomás Torres^1,3,4*^

^1^ Departamento de Química Orgánica, Universidad Autónoma de Madrid, Madrid, 28049, Spain.

^2^ Group for Molecular Engineering of Functional Materials, Institute of Chemical Sciences and Engineering, École Polytechnique Fédérale de Lausanne (EPFL Valais Wallis), Rue de l’Industrie 17, CH-1951 Sion, Switzerland.

^3^ Institute for Advanced Research in Chemical Sciences (IAdChem), Universidad Autónoma de Madrid, Madrid, 28049, Spain.

^4^ IMDEA-Nanociencia, Campus de Cantoblanco, Madrid, 28049, Spain.

[†] These authors contributed equally to this work.

*e-mail: mdkhaja.nazeeruddin@epfl.ch, tomas.torres@uam.es

**Table of content**

[1. Instrumentation and materials 2](#_Toc91959641)

[2. General method to prepare SubPc derivatives 2](#_Toc91959642)

[3. Experimental section of device fabrication and characterization. 3](#_Toc91959643)

[Thin-film perovskite and solar cell fabrication 3](#_Toc91959644)

[Thin-film perovskite and solar cell characterization 3](#_Toc91959645)

# Instrumentation and materials

Nuclear magnetic resonance spectra (^1^H-, ^19^F-, ^11^B-NMR) were recorded on a Bruker AV-300 Bruker DRX-500 spectrometers in the Interdepartmental Investigation Service of UAM. Deuterated solvent employed in each case is indicated in brackets, and its residual peak was used to calibrate the spectra using literature reference δ ppm values.^[[1]](#endnote-1)^ All the experiments were recorded at room temperature.

High-resolution mass spectra (HRMS) were recorded in the Interdepartmental Investigation Service of UAM employing Matrix-Assisted Laser Desorption/Ionization Time-Of-Flight (MALDI-TOF), using a Bruker-Ultraflex-III spectrometer with a Nd:YAG laser operating at 355 nm, for MALDI-TOF. The matrixes and internal references employed are indicated for each spectrum. Infrared Spectra were recorder in solid state on a Bruker Vector 22 spectrophotometer.

Electrochemical measurements were performed on an Autolab PGStat 30 equipment using a threeelectrode configuration system. The measurements were carried out using freshly distilled THF solutions containing 0.1 M tetrabutylammonium hexafluorophosphate (TBAPF_6_) and a concentration of approximately 10^-4^ M of the corresponding compound. A glassy carbon electrode (3 mm diameter) was used as the working electrode, and a platinum wire and an Ag/AgNO_3_ (in CH_3_CN) electrode were employed as the counter and the reference electrodes, respectively. Ferrocene (Fc) was used as external reference and all the potentials were given relative to the Fc/Fc+ couple. Scan rate was 100 mV.s -1. HOMO and LUMO level were calculated using the equation [𝐸_𝐿𝑈𝑀𝑂_=−5.1−𝐸_1/2𝑟𝑒𝑑_ (𝑣𝑠. 𝐹𝑐/𝐹𝑐+) (𝑒𝑉)] and the optical band gap [𝐸_𝐻𝑂𝑀𝑂_=𝐸_𝐿𝑈𝑀𝑂_+𝐸_𝑔,𝑜𝑝𝑡_(𝑒𝑉)].

Ultraviolet and visible (UV-Vis) spectra were recorded using solvents in the spectroscopic grade in the Organic Chemistry Department of UAM employing a JASCO-V660 spectrophotometer. Likewise, fluorescence measurements were carried out with a JASCO-V8600 spectrofluorometer.

The monitoring of the reactions has been carried out by thin layer chromatography (TLC), employing aluminium sheets coated with silica gel type 60 F254 (0.2 mm thick, Merck). The analysis of the TLCs was carried out with an UV lamp of 254 and 365 nm. Purification and separation of the synthesized products was performed by normal-phase column chromatography, using silica gel (230-400 mesh, 0.040-0.063 mm, Merck).

Chemicals were purchased from commercial suppliers and used without further purification. Dry solvents were purchased from commercial suppliers in anhydrous grade or thoroughly dried before use employing standard methods. Solid, hygroscopic reagents were dried in a vacuum oven before use.

The characterization of **Cl-SubPcF_6_, Cl-SubPcF_12_ and Cl-SubPcCl_12_** have been previously reported.^[[2]](#endnote-2)^

# General method to prepare SubPc derivatives

In a 50 mL two-necked round-bottomed flask, equipped with a condenser, magnetic stirrer and rubber seal, a 1.0 M solution of BCl_3_ in *p*-xylene (1.25 mL) was added to the corresponding phthalonitrile 426 mg, 1.25 mmol) in 15 mL of o-DCB under argon atmosphere. The reaction mixture was stirred at 180 ºC reflux for 24 h. The purple solution was allowed to cool to room temperature and flushed with argon. The dark purple reaction slurry was dissolved in toluene/THF 10:1 and passed through a short silica plug. The solvent was removed by vacuum distillation and the resulting dark solid was subjected to column chromatography on silica gel using DCM as an eluent. By washing with methanol, **Cl-SubPcF_6_**, **Cl-SubPcF_12_** and **Cl-SubPcCl_12_** were obtained in a 72%, 55 % and 67% yield, respectively, as a gold, pink and purple solids, respecitvely.


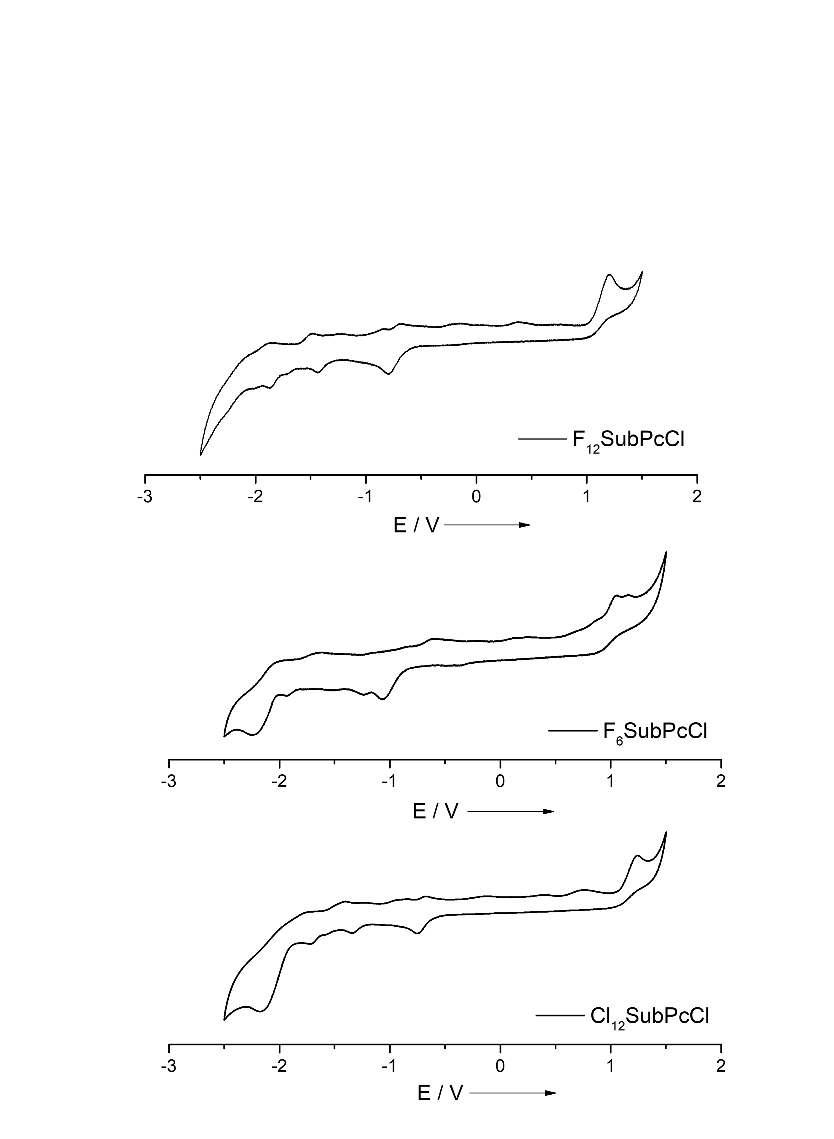


**Figure S1**. Cyclic voltammograms (black lines) and differential pulse voltammograms (red lines) of **Cl-SubPcF_6_, Cl-SubPcF_12_ and Cl-SubPcCl_12_** in THF (V vs. ferrocene/ferrocenium ion couple). Supporting electrolyte, Bu_4_NPF_6_ (0.10 M); working electrode, glassy carbon; counter electrode, platinum wire; reference electrode, Ag/AgNO_3._

# Experimental section of device fabrication and characterization.

## Thin-film perovskite and solar cell fabrication

Laser-etched fluorine-doped tin oxide (FTO)-coated glass substrates (TEC-15AX, NSG group) were cleaned by a sequential sonication treatment (10 min each) in a 2% Helmanex solution, water, deionized water and isopropanol. Then, the clean FTO substrates were exposed to an ultraviolet–O_3_ treatment for 15 min.

A NiO_x_ compact layer was deposited in air by spray pyrolysis deposition (SPD) method using a diluted solution of Nickel acetate acetylacetonate in acetonitrile (0.04 mol/L solution). The film fabrication was carried out at 550 °C and the samples were kept on the hot plate until the substrate temperature reached room temperature. After the NiO_x_ deposition, an ultraviolet–O_3_ treatment for 30 min was performed to enhance the PTAA wetting in the sample and the samples were transferred to a MBraun glovebox (, <0.1 ppm H_2_O, <0.1 ppm O_2_). A thin film of PTAA was deposited from a 0.5 mg/mL solution in toluene, spin-coated at 4,000 r.p.m for 30 s and annealed at 100°C for 10 min.

*Solution-processed perovskite preparation*

Solution-processed CsFAMAPbIBr perovskite layers were deposited on the FTO/NiOx/PTAA samples. The [(FAPbI_3_)_0.87_(MAPbBr_3_)_0.13_]_0.92_(CsPbI_3_)_0.08_ perovskite solution was prepared by dissolving 17.41 mg MABr, 27.02 mg CsI, 57.06 mg PbBr_2_, 178.94 mg FAI and 548.60 mg PbI_2_ in 1 ml of DMF:DMSO mixture (0.78:0.22 v/v). Then, the perovskite layer was prepared by spin coating at 2,000 r.p.m. for 10 s, followed by 5,000 r.p.m. for 30 s. During spinning in the second step, 110 µl of chlorobenzene (antisolvent) was dropped on the sample at the 15 s before finishing the process. The samples were annealed at 100°C for 1 h in an inert atmosphere. Once the samples were cooled down, 100 μL of PEAI (2 mg/mL in isopropanol) was added to the CsFAMAPbIBr during spinning at 5,000 r.p.m and the samples were annealed at 110°C for 30 min in an inert atmosphere.

Once the solution-processed perovskite layer was deposited, thin films of **Cl-SubPcF_6_**, **Cl-SubPcF_12_** and **Cl-SubPcCl_12_** were deposited by thermal evaporation at a deposition rate of 0.1 Å/s at ~175°C, ~135°C and ~240°C, respectively. Prior to its implementation into the PSCs, individual calibration was performed by calibrating the actual perovskite thickness measured by profilometer with the measured thickness monitored by the individual QCM. After the desired SubPc thickness was deposited (0.5, 1 or 2nm), 20 nm of C_60_ (Sigma-Aldrich, 99.9%) followed by 3 nm of bathocuproine (BCP, Sigma-Aldrich, 99.9%) was thermally evaporated. As top electrode, 1 nm Cr and 70 nm Au were deposited by thermal evaporation.

## Thin-film perovskite and solar cell characterization

The thin-film absorbance spectra were recorded with a Lambda 950S spectrophotometer (PerkinElmer, Inc.). The steady-state photoluminescence spectra of the glass/perovskite and glass/perovskite/SubPc thin films were measured upon excitation at 450 nm and recorded using Fluorolog3-22 spectrofluorometer (HORIBA, Ltd.). Time-resolved PL decays were measured with a Time-correlated single-photon counting (TCSPC) Fluorolog, (HORIBA, Ltd.), with an excitation wavelength of 640 nm and detection wavelength of 780 nm (PicoQuant LHD-DC-440 pulsed laser, pulsewidth < 80 ps; F = 9.7 nJ cm^-2^). All the photophysical characterization was performed with the sample illuminated from the front side (perovskite or HTM side). A bi-exponential function was used to fit the TRPL decays.

The *J* – *V* characteristics of the solution- and vacuum-processed PSCs were measured by using a 2400 Keithley system (scan rate: 50 mV s^–1^ and 10 mV voltage step for reverse and forward bias) in combination with a Xe–lamp Oriel sol3A sun simulator (Newport Corporation), previously calibrated to AM1.5G standard conditions by using the reference cell Oriel 91150 V. The devices were measured without light soaking and the illumination area was defined through a shadow mask of 16 mm^2^. The stability test was performed as maximum power tracking under 100 mW cm^−2^ illumination with the Xe–lamp Oriel sol3A sun simulator (Newport Corporation). Prior to the stability test, a *J*-*V* curve was performed to determine the maximum power point (MPP). Once finished, the unencapsulated device was kept under continuously Xe lamp illumination at the MPP during 500 s in ambient atmosphere (25°C, RH<40%). EQE was measured with IQE200B Quantum Efficiency Measurement System (Oriel, Newport). Scanning electron microscopy (SEM) images were recorded by in-lens detector of FEI Teneo Schottky Field Emission SEM at tension of 5 kV. No anti-reflective coating was applied and the devices were measured without encapsulation.

Water-contact angle measurements on the top of perovskite or perovskite/SubPc thin films were measured using the KRUSS DSA100 optical contact angle instrument. Water drops were created at a rate of 0.01 mL/min and a volume of 12 μL with a 0.5 mm needle size. The measurements were conducted using the sessile drop program.

**Figure S2**. a) *V***_OC_**, b) *J*_SC_, c) *FF* and d) PCE distribution of solution-processed *p-i-n* solar cells containing **Cl-SubPcF_6_**, **Cl-SubPcF_12_** and **Cl-SubPcCl_12_**. PCE distribution of reference samples (w/o subphtalocyanine layer) are also presented for comparison.

**Figure S3**. *J–V* curve hysteresis of a) **Cl-SubPcCl_12_** and b) **Cl-SubPcF_12_**.

| **SubPc** | **SubPc** thickness (nm) | PL quenching,  (%) |
| --- | --- | --- |
| **Cl-SubPcF_6_** | 0.5 | 96.6 |
|  | 1 | 91.4 |
|  | 2 | 94.0 |
| **Cl-SubPcF_12_** | 2 | 36.4 |
| **Cl-SubPcCl_12_** | 2 | 76.6 |

**Table S1**. Photoluminescence quenching in % calculated from the corresponding PL emission spectra for perovskite/SubPc samples (perovskite thin film‘s PL emission considered as 100%).

**Figure S4**. *J–V* curve hysteresis with **Cl-SubPcF_6_** layer-thicknesses of a) 0.5 nm and b) 1 nm

**References**

1. [] N. R. Babij, E. O. McCusker, G. T. Whiteker, B. Canturk, N. Choy, L. C. Creemer, C. V. D. Amicis, N. M. Hewlett, P. L. Johnson, J. A. Knobelsdorf, F. Li, B. A. Lorsbach, B. M. Nugent, S. J. Ryan, M. R. Smith and Q. Yang, *Org. Process Res. Dev*., **2016**, *20*, 661. [↑](#endnote-ref-1)
2. [] E. Bukuroshi, A. Mizrahi, Z. Gross and T. P. Bender, *Eur. J. Inorg. Chem.,***2021**, *2021*, 1090-1097 [↑](#endnote-ref-2)
